# Lack of transparency during the COVID-19 pandemic: Nurturing a future and more devastating crisis

**DOI:** 10.1017/ice.2020.271

**Published:** 2020-06-03

**Authors:** Alain Braillon

**Affiliations:** 1 Retired

*“Collaboration is a key part of the success of any organization, executed through a clearly defined vision and mission and based on transparency and constant communication.”* Dinesh Paliwal. (https://en.wikipedia.org/wiki/Dinesh_Paliwal)

*To the Editor—*The editor must be commended for having provided to Rahimi et al^1^ the opportunity to pledge transparency during the COVID-19 crisis, a major issue that has been overlooked in scholarly journals. This issue has two aspects, most concrete.

First, healthcare professionals have faced bullying when speaking out in the media about their real-life experiences of the COVID-19 crisis as they faced basic resources shortage or bureaucratic barriers precluding adequate care or even protecting themselves. In *The New York Times*, Scheiber and Rosenthal reported that nurses and doctors were bullied for speaking out.^2^ Similarly, in the United Kingdom, hospital professionals were gagged for voicing concerns about shortages of equipment to protect against coronavirus.^3^ This occurrence is most frightening because in the United Kingdom the culture of transparency is an old one and has even been strengthened by a comprehensive framework of legal protections: the Employment Rights Act 1996, amended as Public Interest Disclosure Act 1998, and the Defamation Act of 2013. In other European countries, no protections exist for whistleblowers, and the motto seems to be “Silence is golden”!

This issue also extends to scientific committees advising governments. In Great Britain, the government deliberately kept secret the list of participants in its committee of scientific experts.^4^ In France, the Haut Conseil de la Santé Publique (High Council of Public Health), the expert body of the Ministry of Health for the French government, issued 4 dozen reports about COVID-19. As a member, when recruited, I had to sign a form swearing I would respect the “duty of reserve” regarding the content of meetings. This issue is not a theoretical one: I was forced to resign (October 3, 2018) from Public Health France’s scientific committee after a written threat of being sued for such a breach if I refused to resign because whistleblowing by a civil servant is a specific criminal offense in France (Law 83-634, Article 26). This situation contrasts with that the United States where the Lloyd-La Follette Act of 1912 protects civil servants who criticize superiors from official retribution.

In 1998, Söderlund summarized the challenges faced by healthcare systems “protecting against catastrophic illness events,” “improving allocative efficiency and equity of access to services,” and “combating cost escalation,” among many key issues.^5^ Setting transparent and fair rules is a mandatory prerequisite for confidence and effectiveness. Old democracies are deliberately breaching their most basic principle. The crisis is before us.
